# Standardized ethanol extract of *Tinospora crispa* upregulates pro-inflammatory mediators release in LPS-primed U937 human macrophages through stimulation of MAPK, NF-κB and PI3K-Akt signaling networks

**DOI:** 10.1186/s12906-020-03039-7

**Published:** 2020-08-06

**Authors:** Md. Areeful Haque, Ibrahim Jantan, Hemavathy Harikrishnan, Waqas Ahmad

**Affiliations:** 1 Department of Pharmacy, International Islamic University Chittagong, Chittagong, 4318 Bangladesh; 2 Institute of Systems Biology (INBIOSIS), Universiti Kebangsaan Malaysia, 43600 UKM Bangi, Selangor, Malaysia; 3 Department of Pharmacology, School of Medicine, Case Western Reserve University, Cleveland, OH USA; 4 School of Pharmaceutical Sciences, Universiti Sains Malaysia, 11800 USM, Penang, Malaysia

**Keywords:** *Tinospora crispa*, Immunostimulatory actions, Pro-inflammatory cytokines, MAPKs, NF-κB, PI3K-Akt

## Abstract

**Background:**

Immunomodulatory effects of *Tinospora crispa* have been investigated due to its traditional use to treat several inflammatory disorders associated to the immune system. The present study reports the underlying mechanisms involved in the stimulation of 80% ethanol extract of *T. crispa* stems on pro-inflammatory mediators release in lipopolysaccharide (LPS)-primed U937 human macrophages via MyD88-dependent pathways.

**Methods:**

Release of interleukin (IL)-1β and tumor necrosis factor (TNF)-α, and production of prostaglandin E_2_ (PGE_2_) were determined by using enzyme-linked immunosorbent assay (ELISA). Immunoblot technique was executed to determine the activation of MAPKs molecules, NF-κB, PI3K-Akt and cyclooxygenase-2 (COX-2) protein. Determination of pro-inflammatory cytokines and COX-2 relative gene expression levels was by performing the real-time quantitative reverse transcription polymerase chain reaction (qRT-PCR). A reversed-phase HPLC method was developed and validated to standardize the *T. crispa* extract and chemical profiling of its secondary metabolites was performed by LC-MS/MS.

**Results:**

Qualitative and quantitative analyses of chromatographic data indicated that syringin and magnoflorine were found as the major components of the extract. *T. crispa*-treatment prompted activation of NF-κB by enhancing IKKα/β and NF-κB (p65) phosphorylation, and degradation of IκBα. The extract upregulated COX-2 protein expression, release of pro-inflammatory mediators and MAPKs (ERK, p38 and JNK) phosphorylation as well as Akt dose-dependently. *T. crispa* extract also upregulated the upstream signaling adaptor molecules, toll-like receptor 4 (TLR4) and MyD88. *T. crispa*-treatment also upregulated the pro-inflammatory markers mRNA expression.

**Conclusion:**

The results suggested that *T. crispa* extract stimulated the MyD88-dependent signaling pathways by upregulating the various immune inflammatory related parameters.

## Background

Immunomodulators, mostly organic synthetics such as alkylating agents, steroidal and non-steroidal drugs including glucocorticoids as well as biological agents like cytokine inhibitors, interferon inducers, polyclonal and monoclonal antibodies have been presently used to heal immune-related ailments. However, their clinical use as immunomodulators, i.e., immunosuppressants or immunostimulants are with limitations due to their cytotoxicity and severe adverse effects. Thus, immunomodulatory agents with improved safety and efficacy are still in need [1]. The immunomodulating effects of medicinal plants have been attributed to their phytochemicals like polysaccharides, glycosides, flavonoids, alkaloids, lactones, and terpenoids [1–4]. They are broadly used in treating several immune disorders, including inflammatory complaints, autoimmune ailments, as well as cancer. In the last few decades, phytochemicals have attracted great interest as sources of natural immunomodulators, which are less toxic and inexpensive products compared to synthetic therapeutic agents [3].

*Tinospora crispa* (L.) Hook. f. & Thomson belonging to the family Menispermaceae is used widely as complementary and alternative medicine in various parts of the world, especially in Asia and the Pacific [5]. The whole plant, including its stem, roots and leaves are used traditionally to heal various inflammatory disorders related to the immune system. The plant has been reported for use in the treatment of rheumatism, fever, backache, muscle pain, abdominal pain, diabetes, management of internal inflammations, as well as a tonic for managing good health [5, 6]. However, there is lack of scientific investigations to verify these traditional claims.

More than 65 compounds of diverse chemical structures such as alkaloids, flavonoids, lignans, furanoditerpenes, steroids and lactones have been identified in the extract of *T. crispa* and among them, clerodane-type furanoditerpenes are its characteristic constituents [6]. *T. crispa* extract and its fractions have been shown to enhance the intracellular pro-inflammatory cytokines like interleukin (IL)-6, IL-8 and interferon-gamma (IFN-γ) release and expression in vitro as compared to the LPS control in RAW 264.7 cells [7]. *T. crispa* ethanol extract at 100–400 mg/kg exhibited immunostimulatory effects on phagocytosis and chemotaxis of neutrophils, stimulated T- and B-​lymphocytes and T-​lymphocytes subsets CD8^+^ and CD4^+^ proliferation, and prompted the release of T helper type (Th)-1 and Th-2 cytokines like tumour necrosis factor (TNF)-α, IL-2, IL-4 and IFN-γ, [8]. It was also reported that *T. crispa* extract stimulated immune responses in mice by promoting nitric oxide (NO) production in peritoneal macrophages and improved their ability to engulf FITC-labeled *E. coli* in a dose-dependent pattern. It also significantly augmented the serum levels of lysozyme, immunoglobulins (IgM and IgG), myeloperoxidase (MPO) activity, and stimulated sRBS-induced swelling rate of the mice paw in delayed type hypersensitivity (DTH) [9]. In a recent study, *T. crispa* extract and its major constituents were found to stimulate the phagocytic activity and chemotaxis of macrophages and significantly upregulated reactive oxygen species (ROS), NO and pro-inflammatory cytokines production in RAW 264.7 macrophages [10].

Although several investigations on the immunomodulating potential of *T. crispa* have been carried out, the molecular and biochemical mechanisms underlying its effects have not been well investigated. There are a few mechanistic studies on correlating all the signaling events associated with immunomodulation on specific cellular models, especially on the proposed MyD88-dependent signaling network in human macrophages. Recently we reported that magnoflorine, an alkaloid of *T. crispa,* enhanced LPS-primed pro-inflammatory responses in U937 cells and its effects on MyD88-dependent signaling network [11]. However, its immunomodulatory effects, whether stimulating or suppressing, correlate with the polarity and type of extracts used in the experiment. Hence, in the present study, we evaluated the effects of the standardized 80% ethanol extract of *T. crispa* in LPS-stimulated U937 cells on pro-inflammatory signaling molecules release and expression through stimulation of NF-κB, MAPKs and PI3K-Akt signaling pathways.

## Methods

### Chemicals and reagents

Penicillin-streptomycin antibiotic, FBS, PBS, and RPMI-1640 culture medium were attained from Gibco (Grand Island, NY, USA). R & D Systems (Minneapolis, MN, USA) were the supplier of ELISA kits for human IL-1β, TNF-α and PGE_2_. MTT reagent, LPS, RIPA, and DMSO were obtained from Sigma Chemical Co. (St. Louis, MO, USA). Levamisole (purity > 98%) was procured from Cayman Chemical (Ann Arbor, MI, USA). Tocris Biosciences (Bristol, UK) supplied Akt inhibitor (LY294002), p38 inhibitor (SB202190), ERK inhibitor (U0126), JNK inhibitor (SP600125), and NF-κB inhibitor (BAY 11–7082). Pierce (Rockford, IL, USA) supplied 1 × Halt phosphatase and protease inhibitor cocktail. Cell Signaling Technology (Beverly, MA) supplied primary antibodies specific to p-NFκBp65, p-IKKα/β, p-JNK, IκBα, p-IκBα, p-ERK, p-p38, JNK, ERK, p38, COX-2, p-Akt, TRL4 and MyD88 along with anti-rabbit secondary antibody conjugated to horseradish peroxidase and β-actin.

### Plant material and extraction

*T. crispa* stems were collected in the month of June, 2016 from a coastal town of Kuala Terengganu in Malaysia. The plant samples were obtained from the wild and no permission was required to collect the samples. A voucher specimen (UKMB 40178) was identified by Dr. Abdul Latif Mohamad of Faculty of Science and Technology, Universiti Kebangsaan Malaysia (UKM) and deposited at the Herbarium of UKM, Bangi, Malaysia. In brief, air-dried *T. crispa* stems (at 26 ± 2 °C) were ground into powder. Powdered sample (1800 g) was macerated using ethanol (80%) at room temperature for 72 h as previously reported by Haque et al. [11]. The slurry obtained was filtered and the resultant filtrate was evaporated to yield 296 g of brown extract (16.4%).

### HPLC analysis

HPLC analysis of *T. crispa* extract was performed according to the method of Ahmad et al. [9]. Briefly, a stock solution of 10 mg/mL of *T. crispa* in methanol was prepared and the solution was then filtered through PTFE membrane (0.45 μM) (Millipore, Maidstone, Kent, UK). A XBridge™ C18 analytical column (particle size, 5 μm; 4.6 mm × 250 mm) (Waters, Milford, Massachusetts, USA) was employed for the analysis. Quaternary Gradient Module (QGM) (Waters 2535) and photodiode array (PDA) detector (Waters 2998) were used (Waters, Milford, Massachusetts, USA). Solvent A (acetonitrile) and B (trifluoroacetic acid 0.02%) were used as mobile phase. The analysis was performed by elution at 10:90 (A:B) for 10 min followed by 25:75 elution for 25 min at a flow rate of 1.2 mL/min, and detection wavelength was at 254 nm. The extract was qualitatively and quantitatively analyzed using syringin and magnoflorine as standards. Syringin and magnoflorine in the extract were identified by comparing their UV spectra and retention times with those of standards, respectively, run individually.

### Validation of HPLC method

The precision, linearity, limit of quantitation (LOQ) and limit of detection (LOD) were determined for HPLC method validation. The analysis was performed thrice in a day and in three different days for each concentration of sample. The repeatability (intra-assay precision) and intermediate precision (inter-day precision) were carried out. A calibration curve was plotted by using several dilutions of reference standards (62.5–1000 μg/mL) in triplicate. Values from the calibration curves were used to measure linearity (by linear calibration analysis), correlation coefficient (*R*^2^), slope (S) and residual standard deviation (RSD). LOQ and LOD were calculated by following the standard eqs.

### LC-MS/MS analysis

Synergy fusion reverse phase column (C18 polar embedded) (particle size, 3 μm; 100 mm × 2.1 mm) (Phenomenex, CA, USA) and LC-MS/MS (AB Sciex 3200QTrap) with Flexar FX-15 series UHPLC (PerkinElmer, USA) were utilized. Water with 0.1% formic acid and 5 mM ammonium formate (A) and acetonitrile with 0.1% formic acid and 5 mM ammonium formate (B) were used in a gradient program. Sample injection volume was 20 μL. Initially 20–30% B (10 min) was used followed gradually at 250 μL/min to 90–20% B (60 min). The spectra (negative ionization) was obtained by the following settings; full scan - 100-1500 m/z; MS/MS scan - mass range: 50–1500 m/z; capillary voltage: 4500 V; source temperature: 500 °C. The molecular ions accurate mass data was obtained by TOF analyzer. Identification of the constituents were based on comparison of mass fragmentation patterns of each peak with those in the mass spectral library.

### Differentiation induction of U937 cells into macrophages

The U937 cell line (ATCC® CRL1593.2™) was procured from ATCC (Rockville, USA). The cells were cultured in a 75 cm^2^ cell culture flask containing complete culture medium (CCM) and incubated at 37 °C in 5% CO_2_. The CCM was prepared using a plain RPMI-1640 medium together with FBS (10%) and penicillin-streptomycin (1%). The cells were differentiated into macrophages as reported by Haque et al. [11].

### Cell viability assay

Cytotoxicity of *T. crispa* extract was evaluated by MTT assay. The cells (1 × 10^6^ cells/mL) were seeded with several concentrations of the extract in 96 well plates and then kept for 24 h at 37 °C and CO_2_ (5%). After the treatment, 10 μL of 5 mg/mL of MTT solution was added, incubated for 4 h, then quantified by a microplate reader (Tecan Trading AG, Switzerland) [11].

### Cytokines immunoassay

The levels of cytokines in LPS-primed cells after treatment with *T. crispa* extract was investigated by placing 5 × 10^5^ cells/mL of differentiated macrophages into 24 well plates supplemented with 4.68 to 75 μg/mL of *T. crispa* extract or 0.125 to 2 μg/mL of levamisole (positive control) for 2 h, preceding treatment with LPS (1 μg/mL) for 24 h. DuoSet®ELISA System was employed to analyze the effect of the extract and levamisole on TNF-α and IL-1β synthesis following the manufacturer’s directions [11–13].

### Measurement of prostaglandin E_2_

The release of PGE_2_ was quantified using PGE_2_ Assay Kit (R&D Systems, Minneapolis, MN, USA). The differentiated macrophages were treated with varying concentrations of *T. crispa* extract from 4.68 to 75 μg/mL for 2 h followed by LPS stimulation for 24 h. The supernatant was collected and the concentration of PGE_2_ was assessed following manufacture’s recommended instruction [11, 13].

### Quantitative RT-PCR for determination of level of relative gene expression

Innuprep RNA mini kit was used to isolate total RNA from LPS-primed U937 cells to assess the effect of *T. crispa* extract on pro-inflammatory markers (IL-1β, COX-2 and TNF-α) expression. SensiFast cDNA Synthesis kit was employed to synthesize cDNA, following the manufacturer’s recommended procedure. The extracts were kept at − 70 °C until further use. CFX96 Touch Real-Time PCR Detection system, together with SYBR Green RT-PCR Master Mix were utilized for qRT-PCR for mRNA quantification as previously reported [11, 12, 14].

### Immunoblot analysis

Immunoblot technique was performed to determine the protein expression of MAPKs, NF-κB and PI3K-Akt and COX-2 as described previously [11, 13, 14]. Briefly, 4.68 to 75 μg/mL of the extract was mixed with the differentiated cells (1 × 10^6^ cells/mL) for 2 h and then the cells were primed for 30 min with LPS (1 μg/mL) (with the exception for determination of MyD88 and TLR4, 60 min; COX-2, 24 h). Complete protease inhibitor cocktail with RIPA buffer was used to lyse the cells. Centrifugation of the lysate at 13000×g (10 min) was carried out to eliminate insoluble material. The supernatants were taken and kept at − 80 °C. The extracted proteins were separated by 10% SDS-PAGE and transferred onto PVDF membrane, blotted with targeted primary antibodies for overnight. The specific bands were identified by enhanced chemiluminescence reagent and Image Lab™ software was used to analyze the bands intensities. Internal control used was β-actin.

### Downregulation of MAPKs, NF-κB and PI3K/Akt activation

The cells were pretreated with 10 μM of several inhibitors - NF-κB inhibitor (BAY 11–7082), Akt inhibitor (LY294002), JNK inhibitor (SP600125), ERK inhibitor (U0126) and p38 inhibitor (SB202190) along with 75 μg/mL of *T. crispa* extract followed by stimulation with 1 μg/mL of LPS. Then the Western blot and ELISA analyses were employed for determining the protein expression of COX-2 and TNF-α release.

### Statistical analysis

One-way variance analysis and Tukey’s multiple-comparison test were used for calculating the significant differences between treated and untreated groups. GraphPad Prism 6.0 program (San Diego, CA, USA) was employed for statistical analyses and calculations. Every experiment was performed in triplicate, and the results were calculated as mean ± standard error of the mean (SEM). The *p* value less than 0.05 was considered significantly different.

## Results

### HPLC and LC-MS/MS analyses of *T. crispa* extract

**Fig. 1:**
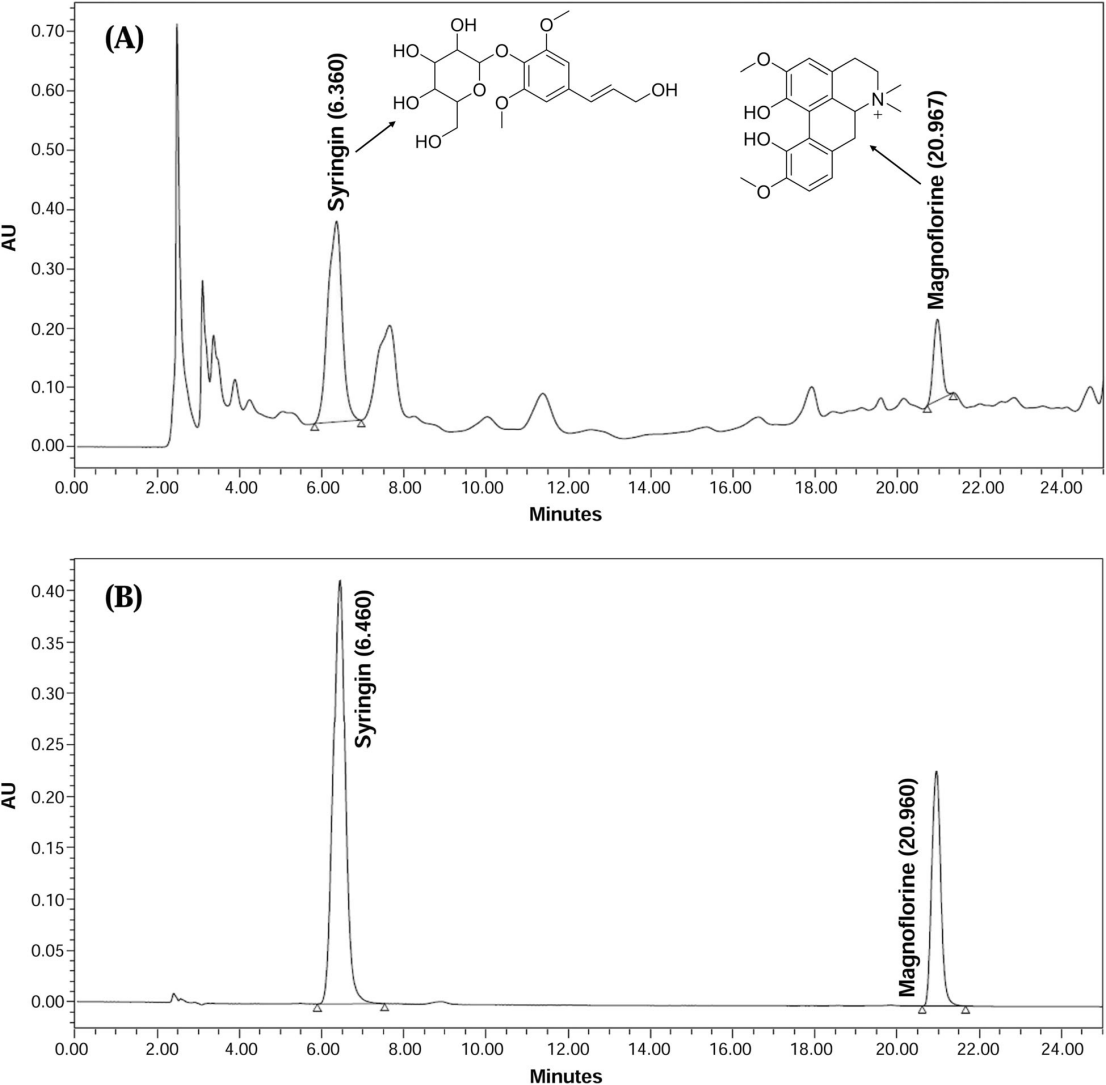
Reverse phase HPLC chromatograms of (**a**) 80% ethanol extract of *T. crispa* standardized to syringin and magnoflorine, and (**b**) standards (syringin and magnoflorine) at the wavelength of 254 nm

Two main peaks representing syringin and magnoflorine with retention times (RT) at 6.360 and 20.967 min, respectively, were identified in the chromatogram of *T. crispa* extract. The peaks was identified by comparing RT of the compounds with those of reference standards, syringin and magnoflorine (Fig. 1). Analysis of the quantitative data indicated that syringin and magnoflorine were found at 466.92 and 281.21 μg/mL, respectively. Calibration curves of syringin and magnoflorine showed a linear curve with R^2^ of 0.998 and 0.997, respectively. For syringin, RSD (%) for intra-day and inter-day precisions of retention time were 0.90 and 1.98%, respectively, while for peak area the precisions were noted as 0.77 and 7.73%, respectively. As for magnoforine, the RSD % values of retention time and peak area for intra-day were 0.24 and 0.58%, respectively, while for inter-day assays the values were 0.011 and 2.78%, respectively. The LOD and LOQ of syringing were 0.0031 and 0.0094 μg/mL, respectively, while for magnoflorine, the values were 0.0078 and 0.024 μg/mL, respectively. LC-MS/MS results for *T. crispa* extract indicated the presence mostly of alkaloids, flavones, terpenes, and phenolic compounds. Some of the secondary metabolites identified were tinorcodiside, tinosponone, columbin, apigenin conjugate, palmatoside, syringin, borapetoside A & C, cordifoliside B & C, palmarin and jateorin. Table 1 shows the retention times of the compounds with their respective molecular ion peaks and MS^2^ fragmentation ions.

**Table 1:** Retention times, MS^2^ fragments of the major compounds present in *Tinospora crispa*

No.	Retention time (min)	Molecular ion peak (M-H)^**−**^	MS^**2**^ fragmentation ions	Tentative compounds identified
**1**	2.256	537^a^	493, 375, 357, 313	Borapetoside A
**2**	4.494	521^a^	341, 311, 297, 253	Cordifoliside B
**3**	5.223	595^a^	549, 485, 432, 355, 322, 269, 159	Apigenin conjugate
**4**	5.727	567^a^	539, 521, 495, 491, 387, 361, 343, 315, 267	Cordifoliside conjugate
**5**	8.195	535^a^	503, 459, 373, 355, 341, 315, 271, 179, 101	Borapetoside C
**6**	10.158	581^a^	536, 504, 373, 355, 315, 179	Borapetoside derivative
**7**	11.170	503^a^	459, 359, 341, 315, 271, 87	Cordifolioside A
**8**	14.376	373^a^	330, 313, 298, 177	Jateorin
**9**	14.422	329^a^	311, 201, 171	3, 3, 0-di-*O*-methyl ellagic acid
**10**	25.438	315^a^	297, 279, 171, 141	Protocatechuic Acid Hexoside
**11**	26.275	373^a^	299, 237	Palmarin
**12**	36.011	521^a^	447, 361, 271, 175	Cordifoliside C
**13**	36.122	491^a^	447, 417, 331, 255, 145	Palmatoside
**14**	38.798	357^a^	339, 295	Columbin
**15**	39.133	329^a^	282	Tinosponone
**16**	40.696	370^a^	352, 326, 308	Syringin
**17**	42.201	395^a^	351, 130	Tinorcodiside
**18**	54.886	635^a^	599, 299	Palmatoside C

^a^base peak

### Effects of *T. crispa* on cell viability

**Fig. 2:**
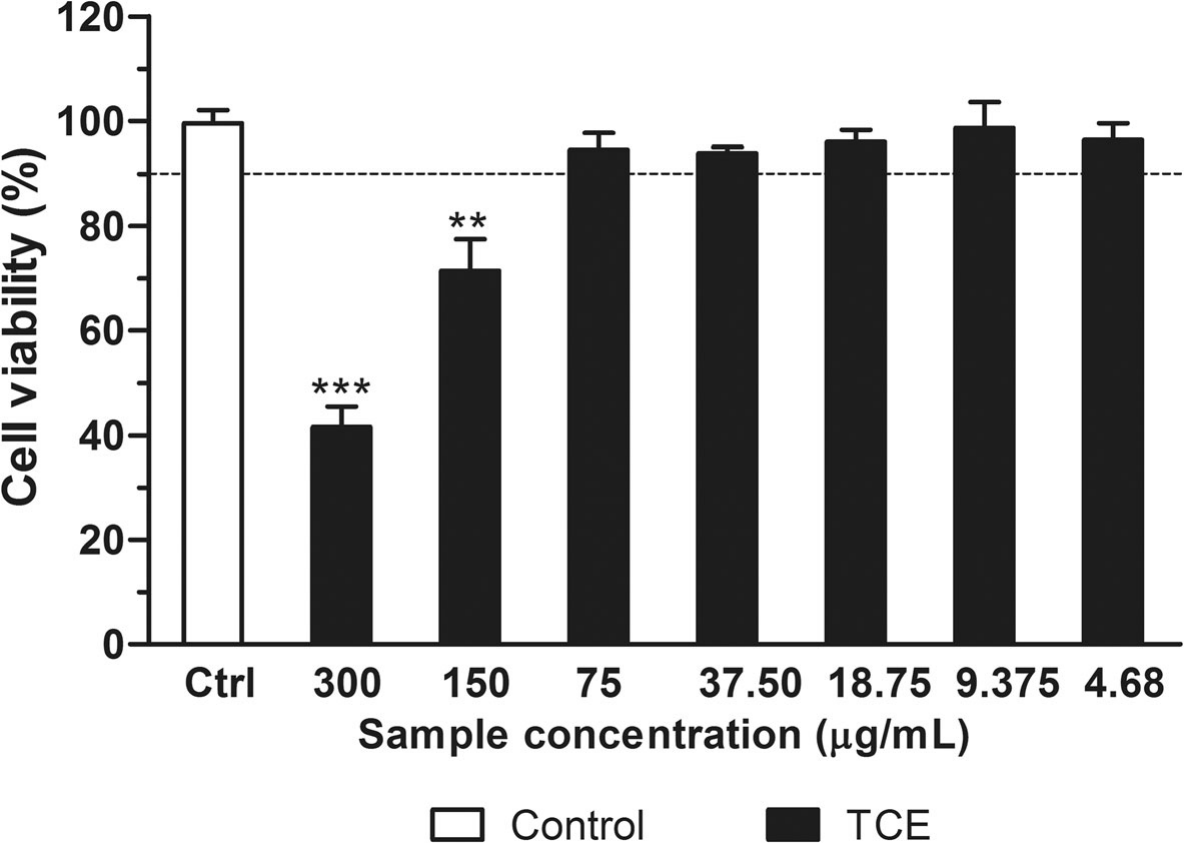
Effect of *T. crispa* extract on viability of differentiated U937 macrophages. Data are presented as mean ± SEM (*n* = 3). **p < 0.01 and ***p < 0.001 denotes significant difference from the control. TCE = 80% ethanol extract of *T. crispa*

To determine the safe concentrations used for this study, U937 cells were treated with 4.68 to 300 μg/mL of 80% ethanol extract of *T. crispa*. As presented in Fig. 2, concentrations at 75 μg/mL and lesser exhibited greater than 90% cell viability. Thus, the safe concentrations used in this experiment were ranging from 4.68 to 75 μg/mL.

### Effects of *T. crispa* on release of pro-inflammatory cytokines and mRNA expression

**Fig. 3:**
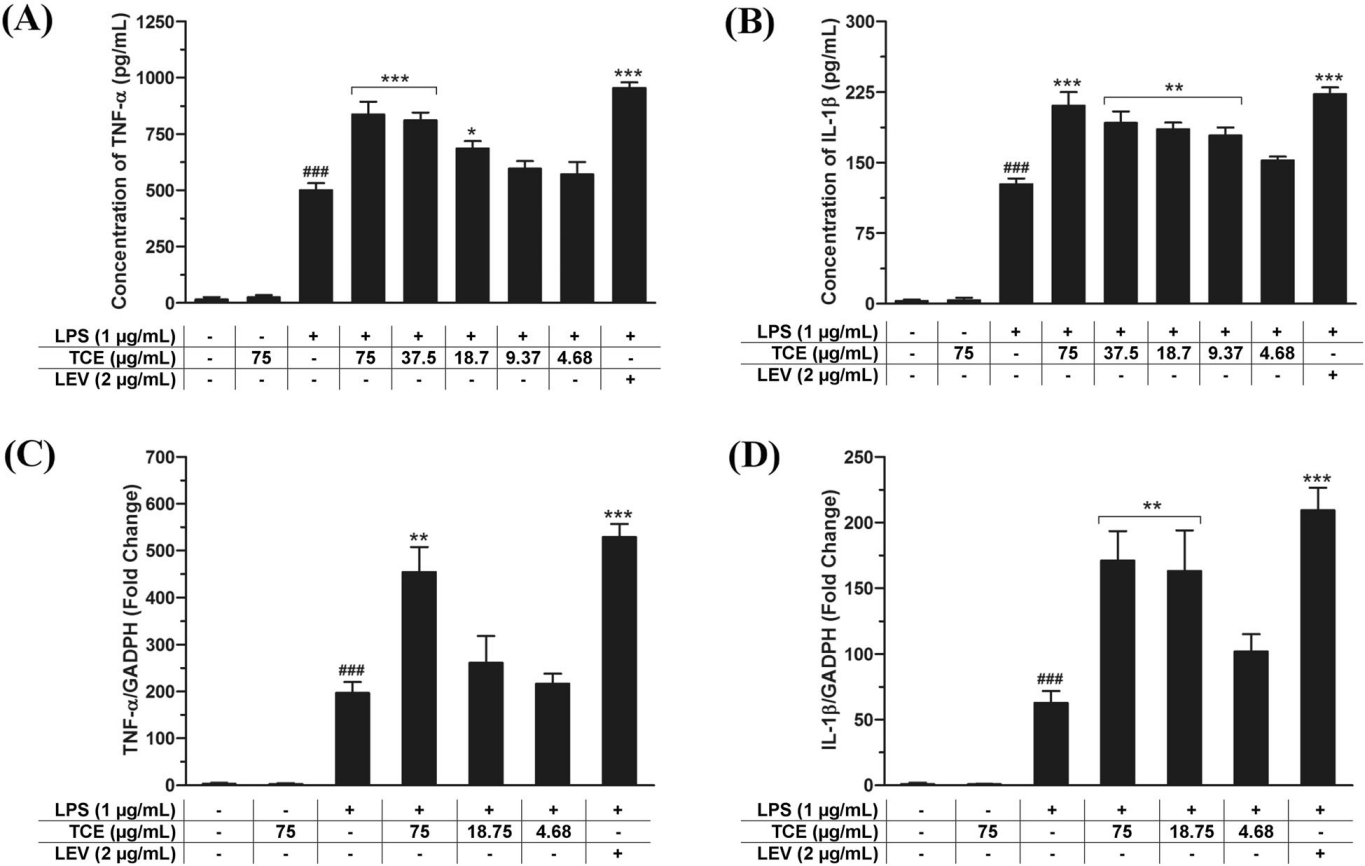
Effects of *T. crispa* on cytokines release in U937 macrophages. **a** and **b** TNF-α and IL-1β production. **c** and **d** TNF-α and IL-1β mRNA expression. ###*p* < 0.001 represents the significant difference from the control. *p < 0.05, **p < 0.01, and ***p < 0.001 represent significance to the LPS alone versus TCE or LEV pretreated. All values are stated as mean ± SEM (*n* = 3). LEV = Levamisole

The pro-inflammatory cytokines are well-known for their intricacy in regulating signaling pathways. TNF-α and IL-1β, which are the predominant pro-inflammatory cytokines are important mediators in the development of many inflammatory diseases. *T. crispa* extract was evaluated on TNF-α and IL-1β release in LPS-primed U937 cells by ELISA. LPS was shown to significantly stimulated cytokines release in the macrophages. *T. crispa* extract augmented the IL-1β and TNF-α release in a dose-dependent pattern (Fig. 3a & b). The EC_50_ values of the extract were 28.09 and 14.68 μg/mL for TNF-α and IL-1β, respectively, whereas levamisole showed EC_50_ values of 0.394 and 0.472 μg/mL for the respective cytokines. Levamisole is a potent immunostimulant as it was reported to be able to upregulate macrophage stimulation and cell-mediated immunity [15]. It is interesting to note that, although both samples enhanced pro-inflammatory cytokines release effectively in the presence of LPS, none of the samples could show any significant enhancements or effects in the absence of LPS.

The effect of *T. crispa* extract was also determined using qRT-PCR to evaluate the cytokines production at the pre-translational level. The mRNA expression of TNF-α and IL-1β was markedly enhanced in LPS-primed U937 cells (196.8 and 62.71 fold, respectively). *T. crispa* extract pre-treatment showed augmentation significantly (*p* < 0.01) at the dose of 75 μg/mL (454.2 fold) for TNF-α as well as at the doses of 18.75 and 75 μg/mL (by 162.9 and 171.1 fold, respectively) for IL-1β mRNA transcription (Fig. 3c & d). Similarly, levamisole at 2 μg/mL emphatically (*p* < 0.001) enhanced the expression of TNF-α gene (528.8 fold). These findings suggest that the upregulation at the protein level of the cytokines was related to the enhancement at the pre-translational level in LPS-primed U937 cells. But the extract without LPS induction could not meaningfully stimulate the IL-1β and TNF-α mRNA expression. Similarly there was insignificant stimulation in untreated control. Thus, treatments with *T. crispa* alone were omitted from further studies.

### Effects of *T. crispa* extract on release of PGE2 and expression of COX-2

**Fig. 4:**
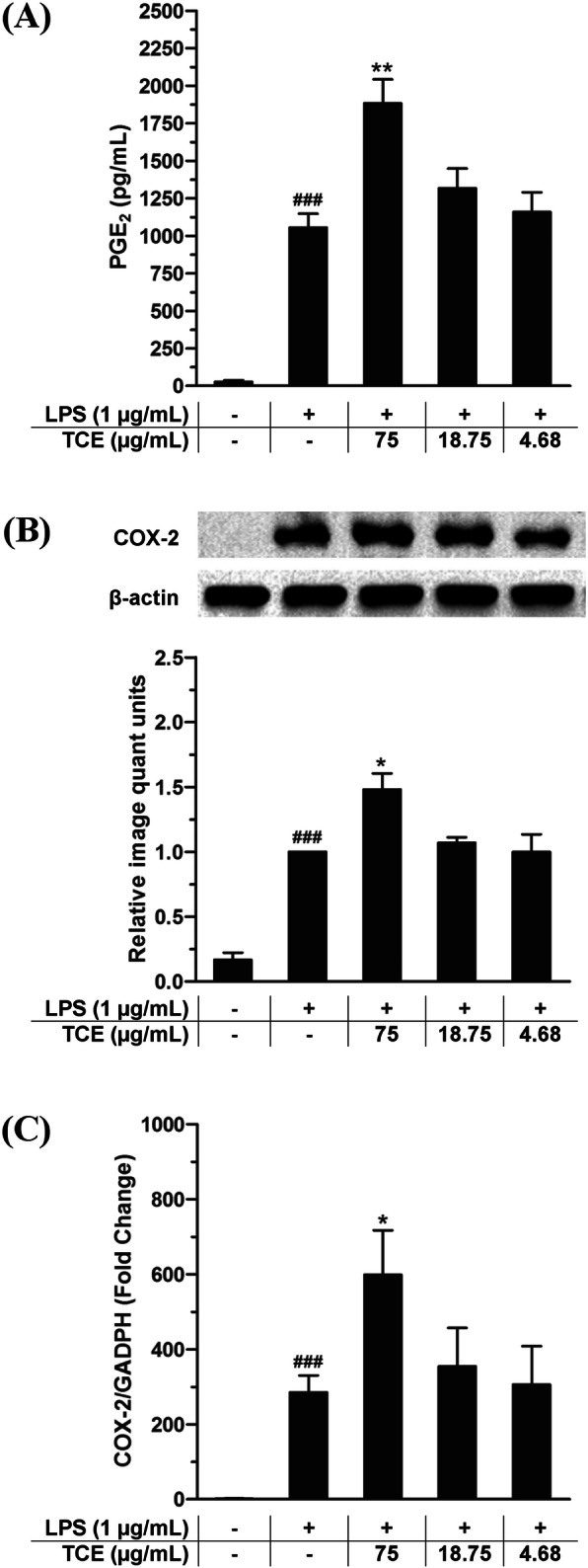
The effects of *T. crispa* on (**a** and **b**) PGE2 production and protein expression of COX-2, (**c**) expression of COX-2 mRNA. ###*p* < 0.001 represents the significant difference from the control. *p < 0.05, **p < 0.01, and ***p < 0.001 represent significance to the LPS alone versus TCE pretreated. All values are stated as mean ± SEM (n = 3)

It was observed that COX-2 protein expression and PGE_2_ production were upregulated in LPS-induced U937 macrophages for 24 h. *T. crispa* extract treatment at 4.68, 18.75, and 75 μg/mL upregulated the protein expression in a dose-dependent pattern. The effect of the extract in LPS-primed macrophages at pre-transitional level was also witnessed. The stimulation of COX-2 mRNA in the cells was improved by 281.9 fold when treated with LPS. Figure 4 shows that *T. crispa* extract uninterruptedly upregulated the expression of COX-2 mRNA in LPS-induced macrophages where the significant upregulation (*p* < 0.05) was observed at the dose of 75 μg/mL (598.1 fold).

### Effects of *T. crispa* on NF-κB activation

**Fig. 5:**
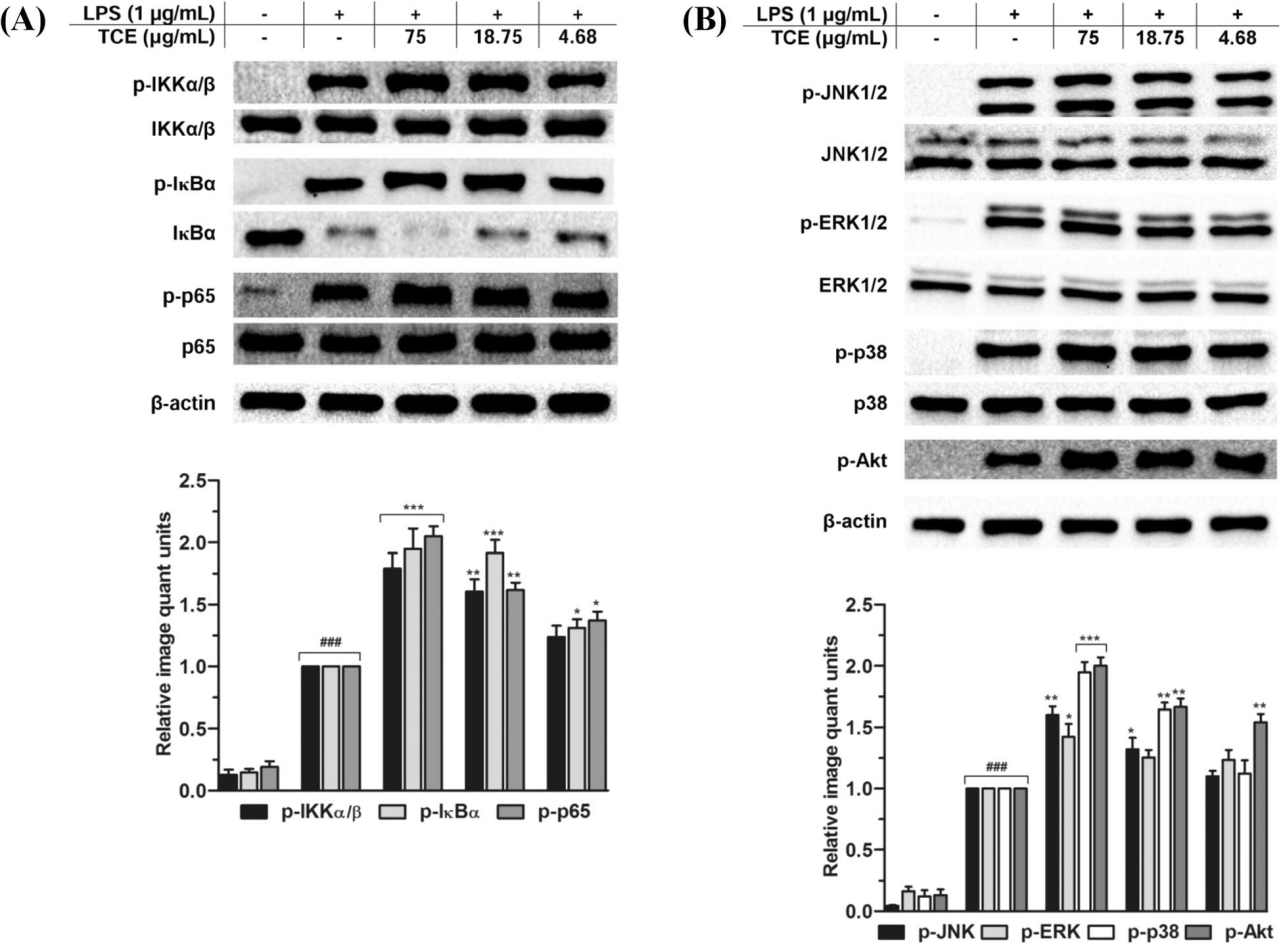
Effects of *T. crispa* on MAPK, NF-κB and PI3K-Akt signaling pathways. Western blots presenting the effects of TCE on (**a**) phosphorylation of IKKα/β, IκBα, and p65 of NF-κB, (**b)** on JNK, ERK, and p38 MAP kinases as well as on phosphorylation of Akt. ###p < 0.001 represents the significant difference from the control. *p < 0.05, **p < 0.01, and ***p < 0.001 represent significance to the LPS alone versus TCE pretreated. All values are stated as mean ± SEM (n = 3)

The effect of *T. crispa* extract on NF-κB activation was evaluated as the latter plays a domineering role in activating numerous immune cells by enhancing many cytokines expressions in accordance with the immune reactions [16]. As depicted in Fig. 5a, the extract expressively augmented the NF-κB(p65) phosphorylation in LPS-primed macrophages. Furthermore, *T. crispa* extract was shown to uphold the phosphorylation and degradation of I-kappa B kinase (I-κBα) level, a significant action for triggering the NF-κB signaling. Subsequently, as I-κBα phosphorylation was interceded by I-κB kinases (IKKs), we too examined whether *T. crispa* extract could endorse the phosphorylation of IKKα/β. Consistent with the immunoblot test, administration of the extract expressively augmented the IKKα/β phosphorylation in a dose-dependent manner. In accordance with the findings of the release of cytokines and mRNA expression, the present results proved that *T. crispa* extract enhanced the release of IL-1β and TNF-α and mRNA expression through upregulating the NF-κB signaling activation.

### Effects of *T. crispa* extract on MAPKs and PI3K-Akt phosphorylation

Besides NF-κB activation, the effects of *T. crispa* extract on phosphorylation of Akt and MAPKs in the LPS-induced U937 cells were also determined. Figure 5b shows that extract treatment enhanced the phosphorylation of Akt dose-dependently and also the phosphorylation of ERK1/2, p38 MAPKs and JNK1/2 without interfering the total levels in LPS-primed macrophages. Notably, the enhancement upon *T. crispa* extract treatment was found prominent in case of Akt phosphorylation (*p* < 0.01) (Fig. 5b).

### Effects of NF-κB, MAPKs, and PI3k/Akt inhibitors

**Fig. 6:**
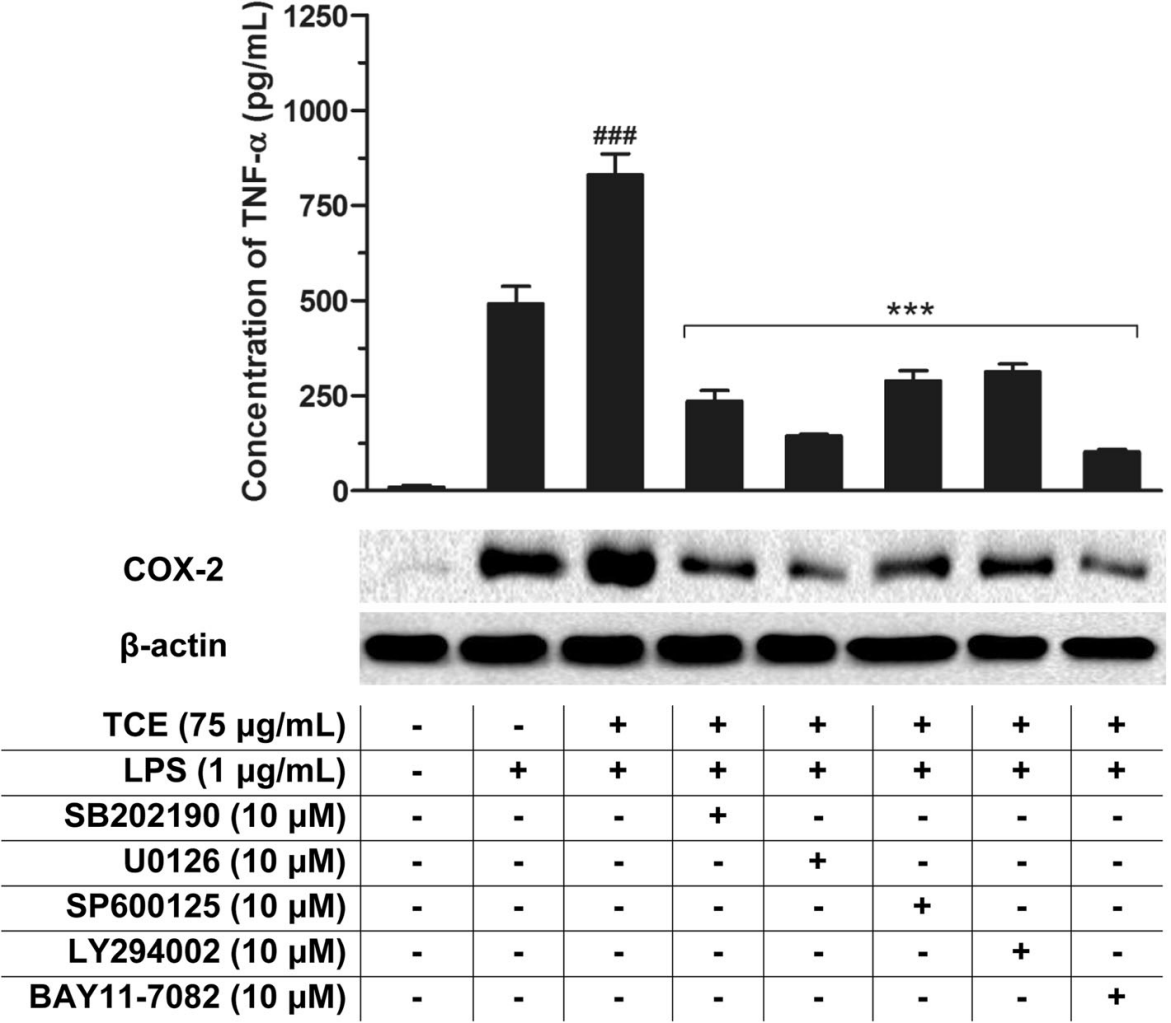
Effects of NF-κB, MAPKs and Akt inhibitors in LPS-activated U937 macrophages; BAY 11–7082 (NF-κB inhibitor), U0126 (ERK inhibitor), SB202190 (p38 inhibitor), SP600125 (JNK inhibitor), LY294002 (Akt inhibitor) on TNF-α production and COX-2 protein expression. ###*p* < 0.001 represents the significant difference from the control. *p < 0.05, **p < 0.01, and ***p < 0.001 represent significance to the TCE pretreated versus inhibitors treatment. All values are stated as mean ± SEM (*n* = 3)

IκBα kinase inhibitor (BAY 11–7082) was used to confirm that NF-κB, MAPKs and PI3K/Akt activation in U937-based pro-inflammatory mediators expression and release were enhanced by *T. crispa* extract. It has been reported that BAY 11–7082 suppressed the activation of NF-κB unambiguously by unnerving the phosphorylation and the subsequent degradation of IκBα [17]. Figure 6 depicts that BAY 11–7082 treatment impeded well the release of COX-2 and cytokine (TNF-α) protein expression that were prompted by the extract in LPS-primed macrophages. Additionally, SP600125, LY294002, SB202190 and U0126, which are the analogous inhibitors of Akt, p38 MAPKs, JNK1/2 and ERK1/2, respectively, also proficiently curbed the release of TNF-α and COX-2 protein expression stimulated by *T. crispa* extract (Fig. 6).

### *T. crispa* extract enhanced TLR4 and MyD88 expression in LPS-induced macrophages

**Fig. 7:**
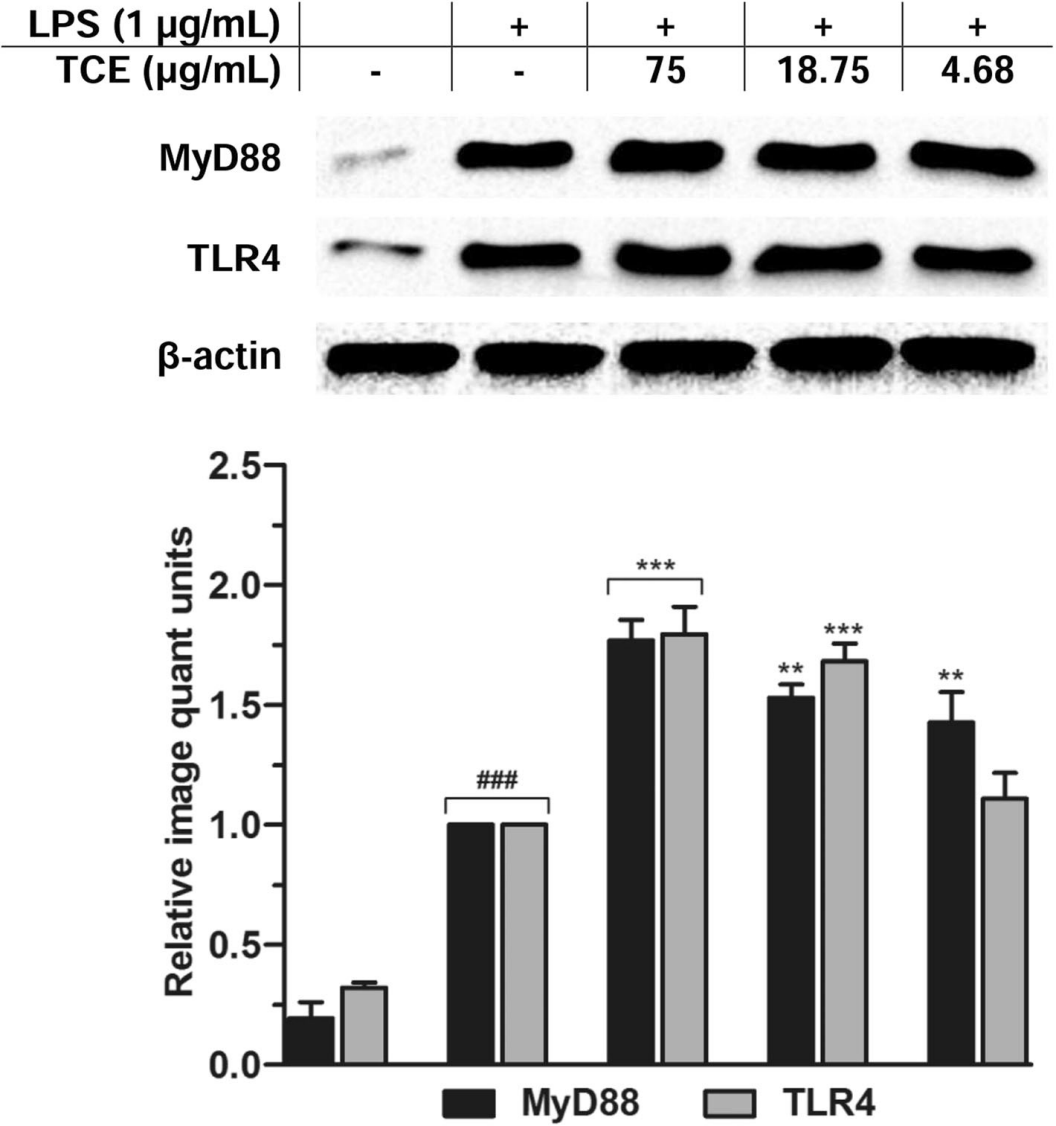
Effects of *T. crispa* on TLR4 and MyD88 expression in U937 macrophages. Respective Western blots presenting the effects of TCE on TLR4 and MyD88 expression. ###*p* < 0.001 represents the significant difference from the control. *p < 0.05, **p < 0.01, and ***p < 0.001 represent significance to the LPS alone versus TCE pretreated. All values are stated as mean ± SEM (*n* = 3)

To determine if these upstream signaling mediators were involved in conniving the activation of MAPKs, PI3K-Akt and NF-κB in LPS-induced U937 cells, the effects of *T. crispa* extract on TLR4 and MyD88 were evaluated. Figure 7 shows that the manifestation of TLR4 and MyD88 upstream signaling adaptor molecules was dose-dependently boosted by *T. crispa* extract. The result was in agreement with the stimulating effects of the extract in LPS-primed U937 cells on the MyD88-dependent pathways.

## Discussion

Macrophages are one of the immune cells that are derived from blood-circulating monocytes. Amongst the monocytes, human U937 cells are widely used as in in vitro experiments as a human macrophage function model [18]. The U937 macrophages display properties similar to the human macrophages while transforming into a macrophage lineage [19]. Macrophages activate the numerous signaling mediators particularly by toll-like receptors (TLRs) to provide the first line of defense. Among the TLRs family, TLR4 (toll-like receptor-4) is the first mammalian TLR to be described and well characterized [20]. Generally, TLR4 is well known as a cellular receptor for bacterial LPS. LPS selectively binds with the TLR4, which further triggers the activation of upstream adaptor molecule known as MyD88 in macrophages. Upon TLR4 activation, MyD88 predominantly triggers the IRAK1 and IRAK4 protein kinases, which embroil the activation of various signaling networks including MAPK, NF-κB, and PI3K-Akt and then initiate mediating various immune markers like pro-inflammatory cytokines, NO, iNOS, PGE_2_, and COX-2.

A controlled inflammatory response is desirable as it provides host defense against infection, tissue-repair response, and adaption to stress and restoration of a homeostatic state [15]. Substances can act as potent immunostimulants if they can promote the immune functions through stimulation of the production of pro-inflammatory mediators in LPS-induced macrophages [21, 22]. Accordingly, the enhancement of the COX-2 protein and gene expression as well as PGE_2_ production by *T. crispa* extract in LPS-primed U937 cells, indicate its immunostimulating capability. In addition, numerous studies revealed that pro-inflammatory cytokines including TNF-α and IL-1β play crucial role in inducing iNOS and COX-2 in LPS-activated macrophages [23]. Hence, the augmenting effects of *T. crispa* extract on the expression of the corresponding pro-inflammatory cytokines suggested that the upregulation of COX-2 expression by *T. crispa* extract might be concomitant with the upregulation of the pro-inflammatory cytokines expression in macrophages. It is interesting to note in this study NO could not be detected in measurable amount in the LPS-stimulated U937 cells. This is consistent with previous reports which suggested that tetrahydrobiopterin (BH4), an essential cofactor for NO synthesis, is lacking in U937 cells [13, 24, 25].

The effect of *T. crispa* extract on the activation of transcription factor NF-κB was investigated as it controls numerous gene regulations including the respective pro-inflammatory markers. NF-κB is a major drug target to treat diverse types of diseases as it plays important role in several disease states [26–28]. In a normal cell, NF-κB activities are discretely regulated by proteolysis and phosphorylation of several NF-κB inhibitors such as I kappa B (IκB) proteins. NF-κB dimer (p65/p50) in a resting cell normally binds to the inhibitory IκB protein in the cytoplasm. The IκB protein rapidly undergoes phosphorylation and degradation by the proteosomal pathway in response to LPS or the pro-inflammatory stimuli [29, 30]. Hence, the free NF-κB releases and translocates into the nucleus of cell where it interacts with targeted genes to form specific binding with the promoter sites. This will lead to stimulation of the genes transcription for mediating copious immune markers including respective pro-inflammatory mediators [31, 32]. From this investigation, we observed that *T. crispa* prompted the NF-κB (p65) phosphorylation followed by enhancing the IκBα phosphorylation and degradation. Moreover, *T. crispa* extract treatment remarkably augmented the IKKα/β phosphorylation without interfering the total level. Expectedly, these results are in agreement with the significant augmentation exhibited by *T. crispa* extract on the respective release of the pro-inflammatory markers and their mRNA expression.

Apart from MAPKs, NF-κB and PI3K-Akt signaling pathways also mediate central role during numerous cellular and molecular processes related to immune response as well as corresponding NF-κB activation. We witnessed from our investigation that *T. crispa* extract successively augmented the phosphorylation of three MAPKs (JNK, ERK and p38) and Akt without interfering the total protein level. The findings were further justified from the effects of specific inhibitors of the MAPKs, NF-κB and PI3K-Akt pathways. The specific inhibitors blocked the *T. crispa* extract-triggered release and expression of COX-2 and TNF-α. This indicates that MAPKs, NF-κB and PI3K-Akt networks possessed major roles in *T. crispa* extract-triggered pro-inflammatory responses and macrophage activation.

TLR4 is a critical signaling receptor for LPS, possesses vital role in mediating innate and acquired immunity. They contribute in innate immunity by identifying PAMPs of various microbial agents [33]. LPS stimulation of TLR4 sequentially triggers the intracellular pathways through the receptor dimerization and recruitment of adaptor molecule, MyD88. The TLR4 dimerization generally activates two leading pathways i.e., MyD88-dependent and MyD88-independent pathways. The MyD88-dependent pathway is triggered once MyD88 associates with TIR (toll/IL-1 receptor) and then forms complex with IRAK-4 that facilitate IRAK-1 to recruit TRAF-6. The complex of IRAK1-TRAF6 phosphorylates TAK-1, which further leads to the regulation of prospective NF-κB, MAPK, and PI3K-Akt signaling pathways [34–36]. This study showed that treatment with *T. crispa* also stimulated the activation of MyD88 and TLR4, which is consistent with the outcomes of the previous studies. On this perspective, it is suggested that *T. crispa* extract targets the MyD88 and TLR4 signaling molecules to mediate the actuation of MAPKs, NF-κB and PI3K-Akt networks and hence, promoting the synthesis and release of pro-inflammatory mediators to enhance immune function.

Pleiotropic or multi-targeted effect is commonly found in numerous medicinal plants and natural immunomodulaors. The stimulatory or inhibitory effects of plant samples on the immune systems in in vitro or in vivo experiments correlate with the different types of extracts or fractions used. The chemical components contributing to the effects should be identified as its bioactive chemical markers. In our present investigation, we witnessed that *T. crispa* extract could stimulate the immune system and acted on multiple targets. The results of this in vitro study would be useful for future mechanistic studies of the immunostimulatory effects of *T. crispa*, to justify its ethnomedicinal claim to treat immune inflammatory related diseases. Besides, from the HPLC and LC-MS/MS analyses of the extract it can be estimated that the prevailing immunoaugmenting potential of *T. crispa* extract might be due to the presence of several active chemical markers like magnoflorine and syringin in it.

## Conclusion

**Fig. 8:**
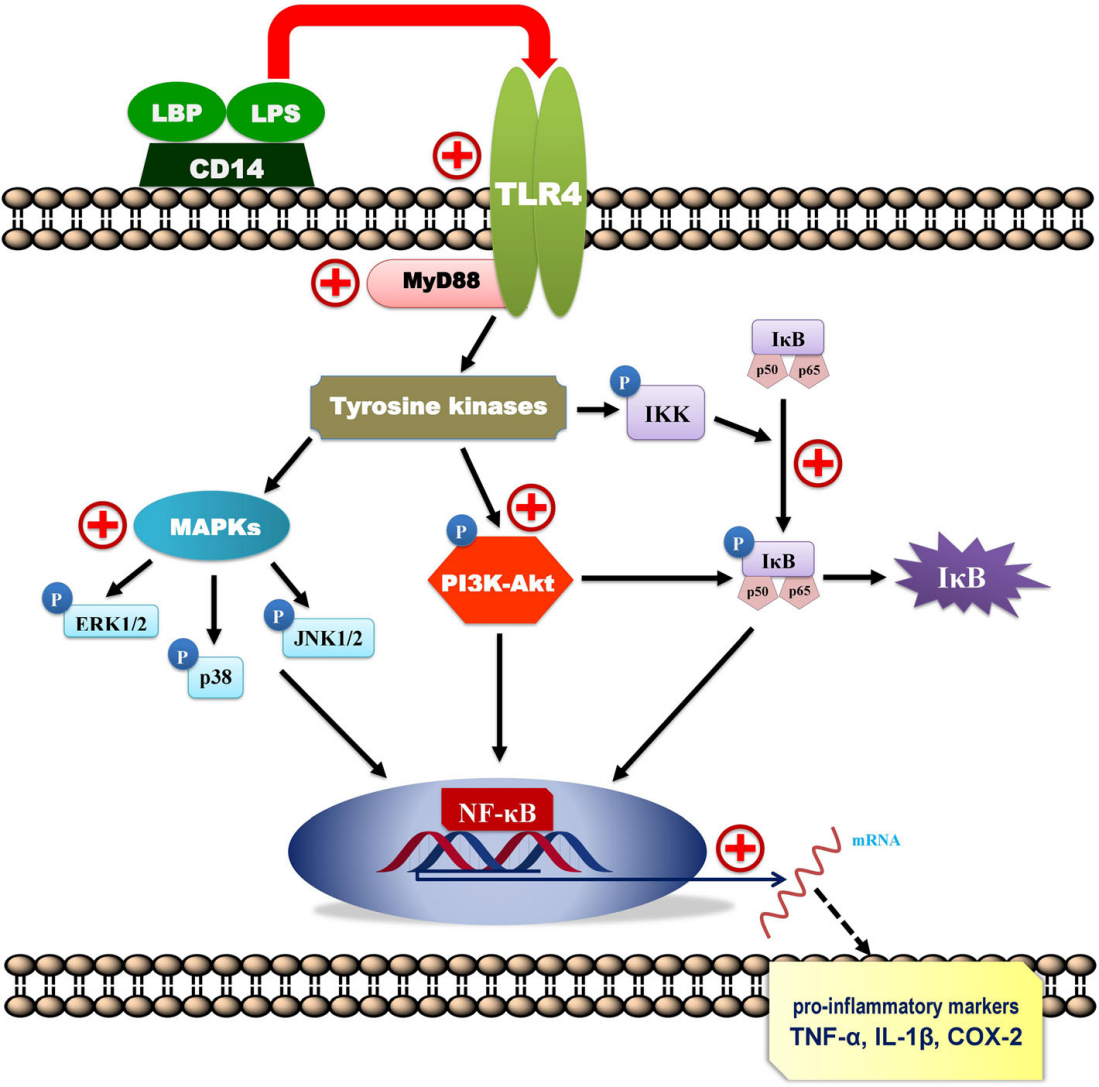
Schematic illustrations of putative signaling mechanisms of *T. crispa* in upregulating the MAPKs, NF-κB and PI3K-Akt signaling networks. The plus (+) indicates the possible *T. crispa* stimulation of the pathways

Taken together, from the above investigation it was demonstrated that *T. crispa* extract stimulated the pro-inflammatory responses through augmenting the respective mediators in LPS-activated human macrophages. Moreover, *T. crispa* extract enhanced the macrophage function followed by the LPS-induced activation of corresponding MyD88-dependent signaling pathways remarkably (Fig. 8). The presence of several active chemical markers as revealed in the quantitative and qualitative analysis might be responsible for these potent effects. Hence, extensive phytochemical studies have to be carried out to isolate and identify active metabolites contributing to its immunostimulating properties followed by planned immunophamacological screenings. Besides, more mechanistic investigations are recommended including effects on more signaling pathways and molecular markers such as kinase activities of Syk, Src, and IRAK-1, the promoter activity of AP-1, and CREB on the analogous. Effects of the plant on other cell lines such as human THP-1 cell line and PBMC-derived macrophages are also recommended. Further studies have to be pursued, which include prospective pharmacokinetic study, bioavailability, toxicological and in vivo delivery studies prior to clinical studies to develop the plant into a potent immunostimulator including as dietary supplements and nutraceuticals.

## Data Availability

Available from the corresponding author on reasonable request.

## Abbreviations

COX-2: cyclooxygenase-2
DTH: delayed type hypersensitivity
IgM and IgG: immunoglobulins M and G
IL-1β: interleukin-1 beta
LOD: limit of detection
LOQ: limit of quantitation
LPS: lipopolysaccharide
MAPKs: mitogen activated protein kinases
MyD88: myeloid differentiation primary response gene 88
MPO: myeloperoxidase
NF-κB: nuclear factor-kappa B
NO: nitric oxide
PI3K-Akt: phosphatidylinositol 3-kinase
PGE2: prostaglandin E2
ROS: reactive oxygen species
(qRT-PCR): real-time quantitative reverse transcription polymerase chain reaction
sRBC: sheep red blood cells
TNF-α: tumor necrosis factor alpha
Th-1 and Th-2: T helper type-1 and type 2
TLR4: toll-like receptor 4
